# Lumbar Intraspinal Calcium Pyrophosphate Deposition: A Comprehensive Case Study

**DOI:** 10.1177/11795735251347335

**Published:** 2025-06-06

**Authors:** Juan M. López-Navarro, Diego A. Sandoval-Lopez, Pavle Popovic, Vasileios Karantzoulis, Zeid Bittar, Edgar Santos, Farzam Vazifehdan

**Affiliations:** 1 Department of Neurosurgery, School of Medicine and Health Sciences, 11233 Carl von Ossietzky Universität Oldenburg, Oldenburg, Germany; 2 Spine Center Stuttgart, Paulinenhilfe, 39928 Diakonie-Klinikum Stuttgart, Stuttgart, Germany; 3 Clinic for Diagnostic and Interventional Radiology, 39928 Diakonie-Klinikum Stuttgart, Stuttgart, Germany; 4 BAG for Pathology and Molecular Pathology, Stuttgart, Germany

**Keywords:** calcium pyrophosphate deposition disease, calcium pyrophosphate crystals, chondrocalcinosis, lumbar spine, spinal canal stenosis, posterior longitudinal ligament

## Abstract

**Introduction:**

Calcium pyrophosphate deposition (CPPD) disease is characterized by calcium pyrophosphate crystals in hyaline and fibrocartilage. Chondrocalcinosis, a radiographic hallmark for CPPD, becomes more prevalent with age. Although CPPD mainly targets peripheral joints, spinal involvement, affecting intervertebral discs and spinal ligaments, is less common but significant, seen in 24.3% of hospitalized patients with CPPD disease. This report describes a rare case of spinal CPPD causing spinal canal stenosis in the lumbar region.

**Case Description:**

A 79-year-old woman with a 3-year history of low back pain presented with severe left-sided pain and mobility impairment. Initial examination showed lumbar tenderness and normal muscle strength. Computed tomography (CT) and magnetic resonance imaging scans revealed a calcified extradural mass occupying the anterior portion of the lumbar spinal canal, most likely associated with the posterior longitudinal ligament. The patient underwent L3-L5 hemilaminectomies and dorsal spondylodesis, removing a whitish intraspinal mass. Histopathology confirmed CPPD. Post-surgery, the patient experienced initial pain relief but required emergency surgery due to complications. Over the next year, her mobility and pain improved significantly.

**Discussion:**

Spinal CPPD manifests with varied clinical presentations, complicating diagnosis. Imaging reveals calcifications ranging from deposits to mass-like lesions causing compression. CT provides detailed visualization of characteristic calcifications, aiding in diagnosis, while histopathology remains the gold standard. Multidisciplinary collaboration is vital for accurate diagnosis and optimal management.

## Introduction

Calcium pyrophosphate deposition (CPPD) disease, which commonly manifests as symptomatic arthritis affecting mainly middle-aged and elderly individuals, is characterised by the accumulation of calcium pyrophosphate (CPP) crystals within hyaline and fibrocartilage tissues, alongside periarticular structures.^1,2^ The term “chondrocalcinosis” denotes a typical radiographic finding marked by linear or punctate opacities, which correlates with CPPD disease but does not invariably indicate clinical arthritis.^3,4^ The prevalence of chondrocalcinosis escalates with age, ranging from 4% to ≥ 10% among older adults; however, recent studies using ultrasound imaging suggest that the actual prevalence of CPPD may be significantly higher. In a cross-sectional study of consecutive patients with knee pain, ultrasound detected CPPD in fibrocartilage in up to 46.7% of individuals over 80 years of age.^5^ The symptomatic phenotypes principally include acute inflammatory arthritis (formerly known as pseudogout), chronic inflammatory arthritis and osteoarthritis with crystal deposition.^3,6^

While CPPD disease primarily targets peripheral joints, spinal manifestations are less common, mainly affecting intervertebral discs and ligaments, with the ligamentum flavum, transverse, alar, and posterior longitudinal ligaments being the most frequently involved.^3,7^ However, recent findings reveal spinal involvement in 24.3% of hospitalised patients with CPPD disease, predominantly affecting the cervical and lumbar regions.^8^ Conversely, postoperative prevalence rates of spinal CPPD, determined from histopathological specimens of patients initially presenting with chronic lower back pain, range from 14.7% to 57.8%.^9^ This suggests a probable underestimation of the spinal involvement in CPPD disease.

In this comprehensive case report, we present a case of spinal CPPD causing severe canal stenosis due to an accumulation of CPP crystals in the anterior portion of the lumbar spinal canal, most likely associated with the posterior longitudinal ligament. This case is notable due to the unusual intraspinal location and the extent of crystal deposition, which led to marked neurological symptoms and posed significant diagnostic challenges. We thoroughly examine the diagnostic process, detailing clinical presentation, imaging findings, and histopathological correlation.

## Case Description

### Initial Presentation and Clinical Findings

A 79-year-old woman with a history of chronic back pain over the past 3 years presented with a recent 4-week worsening of symptoms, characterized by severe low back pain radiating to the left leg and foot. This significantly impaired her mobility and quality of life. Physical examination revealed limited mobility, normal muscle strength and reflexes, and significant lumbar tenderness. Her medical history included monoclonal gammopathy of undetermined significance with IgG kappa light chains, vitamin D deficiency and chronic knee and finger arthritis. Blood tests were normal.

### Imaging and Diagnostic Work-Up

**Figure 1.**
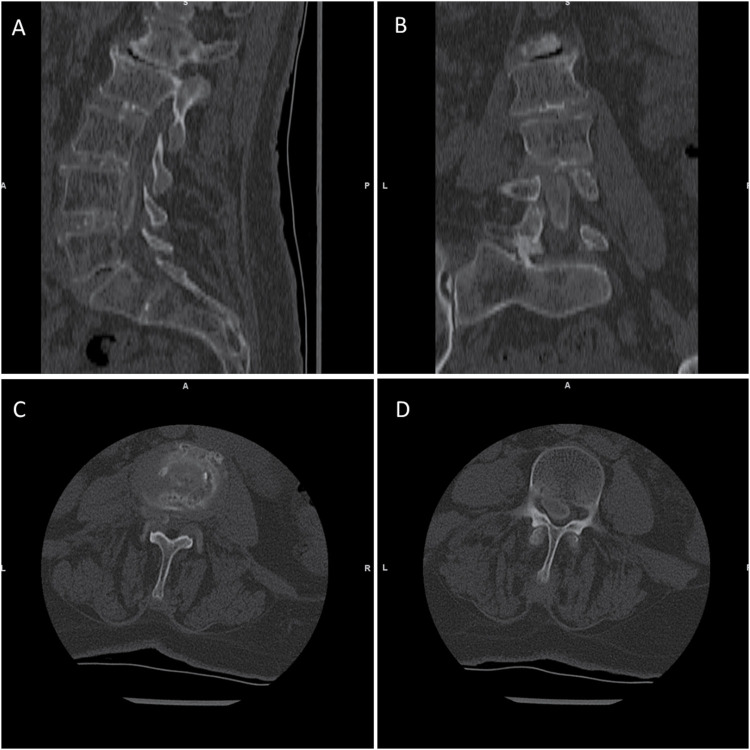
Preoperative Lumbar Spine CT Scan Bone Window. (A) The Sagittal Image Reveals Linear and Punctate Calcifications within the Intervertebral Discs From L2/3 to L4/5, Appearing less Dense than Cortical Bone. Disc Bulging and Herniations are Observed at the Endplates of L2 and L3, as Well as L3/4. Erosive Changes are Noted in the L2/3 Endplates, Accompanied by Moderate Ventral and Dorsal Spondylophyte Formations and Moderate Ventral Spondylophytes on the Superior Endplate of L4. In the Anterior Spinal Canal, Elongated, Spindle-Shaped to Lobulated, Inhomogeneous Calcifications Extend Between L2 and L5, With Resulting Severe Spinal Stenosis at L3/4 and L4/5 (B) the Coronal Image Demonstrates the Calcified Intraspinal Mass Predominantly Located on the Left Side, with its Widest Transverse Diameter at the Level of the L4 Vertebral Body. (C) Axial Imaging at the L3-L4 Intervertebral Disc Shows Well-Defined Linear, and Punctate Calcifications, Less Dense than Cortical Bone. (D) The Axial Image at the L4 Vertebral Body Reveals the Presence of the Calcified Intraspinal Mass, Measuring Approximately 18 mm × 11 mm in its Largest Axial Diameter, Occupying the Anterior Spinal Canal and Most Likely Associated with the Posterior Longitudinal Ligament.

Spinal computed tomography (CT) and magnetic resonance imaging (MRI) were performed shortly after admission to evaluate the source of the patient’s symptoms. CT imaging was particularly instrumental in identifying mineralized lesions; it revealed pronounced punctate and linear calcifications in the intervertebral disc from L2/3 to L4/5 (Figure 1). Additionally, multisegmental disc bulging and intervertebral disc herniations were observed at the endplates of L2 and L3, as well as at L3/4. Erosive changes in the endplates were seen at L2/3, accompanied by moderate ventral and dorsal spondylophyte formations and moderate ventral spondylophytes on the superior endplate of L4.

In the anterior spinal canal, CT clearly depicted elongated, spindle-shaped to lobulated, inhomogeneous calcifications from L2 to L5, most likely associated with the posterior longitudinal ligament. Notably, these calcifications extended focally into the intervertebral foramen, particularly at L4/5 on the left, suggesting involvement of the deep layer of the posterior longitudinal ligament, which is centrally connected to the annulus fibrosus and extends foraminally. These calcified masses contributed to severe central spinal canal stenosis, with the most pronounced narrowing at L3/4 and L4/5, measuring approximately 18 mm × 11 mm in axial diameter.

**Figure 2.**
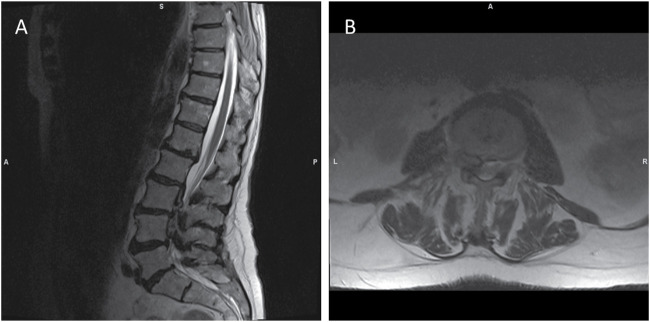
Preoperative Non-Contrast T2-Weighted MRI Sequence. (A) The Sagittal Image Reveals Multisegmental Disc Bulging and Intervertebral Disc Herniations at the Endplates of L2 and L3, as Well as at L3/4. Degenerative Changes are Observed at the L1/L2 and L2/L3 Endplates, With Activated Osteochondrosis Noted at L1/L2. Severe Spinal Canal Stenosis is Evident at L3-L5, with the Most Pronounced Narrowing Along the Posterior Edge of L4 and at L4/5. (B) Axial Imaging at L4 Demonstrates an Extradural Mass Occupying the Anterior Spinal Canal, Resulting in Significant Radicular Compression and Severe Canal Stenosis. The Intraspinal Mass Appears Hyperintense and is Surrounded by a Hypointense Rim.

MRI added essential complementary information by better characterizing soft tissue structures and the nature of the intraspinal mass. In T2-weighted images, the mass appeared centrally hyperintense with a surrounding hypointense rim, while it was hypointense on T1-weighted images (Figure 2). MRI confirmed the CT-based suspicion of severe spinal canal stenosis, with compression most evident along the posterior edge of L4 and at L4/5. MRI also revealed additional degenerative changes in the endplates and vertebral attachment structures, consistent with the CT findings, and identified activated osteochondrosis at L1/2.

### Surgical Intervention

Due to the severity of stenosis and progressive neurological symptoms, the patient underwent left-sided hemilaminectomies from L3 to L5 with dorsal spondylodesis. Intraoperatively, a whitish, partially fluid intraspinal mass with granular content was identified and debulked. Dense inflammatory adhesions with the dura were resected, and specimens were collected for microbiological and histopathological evaluation.

### Postoperative Complication and Re-Intervention

Post-surgery, the patient experienced significant relief from bilateral lower-extremity pain and paresthesias, with moderate improvement in back pain. However, within the first 24 h, she developed acute neurological deterioration, including new-onset left leg paresis, urinary retention, and severe radicular pain. Emergency imaging revealed a postoperative epidural hematoma extending from L3 to L5 and evidence of a fractured L4 pedicle with signs of spinal canal narrowing. These findings explained the rapid clinical decline. A second surgery was performed urgently, involving evacuation of the hematoma, removal of bony fragments, bilateral decompression (extended over-the-top to the contralateral side), and neurolysis of the L3 and L4 nerve roots.

### Histopathological Findings

**Figure 3.**
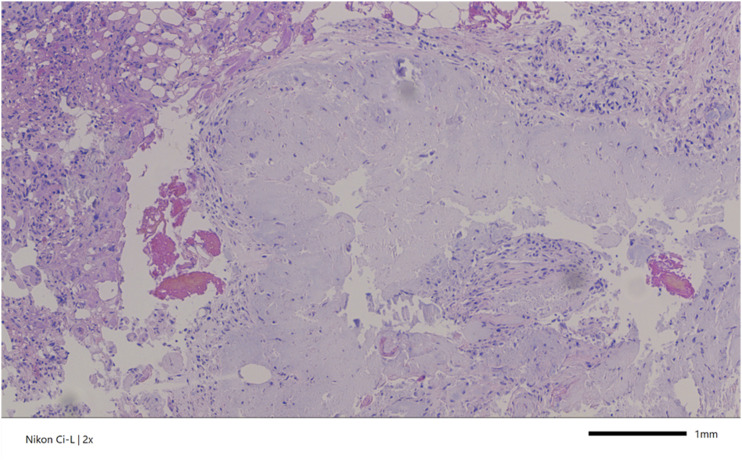
Histopathological Finding of Calcium Pyrophosphate Crystals. This Histological Image, Stained with Hematoxylin and Eosin (H&E), Displays Connective and Cartilaginous Tissue with Deposits of Dissolved Crystals, Indicative of Chondrocalcinosis. The Surrounding Tissue Exhibits Chronic, Granulomatous Inflammation with Areas of Resorption and Numerous Giant Cells. Dystrophic Calcifications are also Evident.

The macroscopic examination involved a tissue sample measuring 1 cm in diameter. The diagnosis and evaluation were based on various staining techniques, including Hematoxylin and Eosin, Periodic Acid-Schiff, Elastica van Gieson, Alcian Blue, and polarisation. Histologically, the tissue consisted of connective and cartilaginous tissue with deposits of dissolved crystals (Figure 3). These deposits were surrounded by chronic inflammation characterised by granulation and, in some areas, resorption, with numerous giant cells present. Additionally, dystrophic calcifications were observed. The presence of double-refracting, weakly birefringent crystals on polarized light microscopy, along with the Alcian Blue stain positivity, was diagnostic of CPPD disease. Thus, the integration of radiological and pathological findings was critical in establishing a definitive diagnosis.

### Recovery and Follow-Up

After initial recovery, the patient began inpatient neurorehabilitation approximately 2 weeks following discharge. At the first outpatient follow-up 3 months postoperatively, the patient reported significant improvements in her mobility and overall recovery. During her physical examination, it was observed to walk with a small, steady pace without the need for a walker. The strength assessment revealed that her left hip flexors and knee extensors had a strength rating of 3/5. In contrast, all other major muscle groups exhibited full strength, rated at 5/5, on both sides. By the second follow-up, 8 months postoperatively and without the need for analgesic medication, muscle strength had further improved to 4/5, and back pain was now only triggered by certain movements. Nearly a year post-surgery, the patient reports persistent mild low back pain, with no apparent muscle weakness or signs of radiculopathy. Fourteen months after spine surgery, the patient underwent surgery on the left knee for isolated left patellofemoral osteoarthritis. The procedure included a patellofemoral joint replacement, partial synovectomy, and osteophyte resection. The knee X-ray showed no evidence of chondrocalcinosis and pathology results from the synovial tissue of the left knee indicated low-grade synovitis and pronounced siderosis, with no evidence of crystalopathy.

## Discussion

The clinical presentations of spinal CPPD vary widely, often complicating diagnosis.^9^ Crowned dens syndrome, caused by CPP deposits around the C2 vertebra, presents with acute neck pain, fever, and elevated inflammatory markers.^3,10^ Lumbar CPPD, less described, may involve acute, subacute, or chronic spinal pain, sometimes mimicking conditions like infectious spondylodiscitis,^11,12^ facet joint septic arthritis,^13^ epidural abscess, hematoma,^14^ or degenerative spine disorders.^9^ The discussed case involved a calcified extradural mass occupy the anterior spinal canal, causing severe stenosis, chronic back pain, and radiculopathy. Mass-like epidural or intradural CPPD lesions in the lumbar spine are seldom reported in the literature. Similar to our case, Gewolb et al^7^ described an intradural CPPD mass at L3-L4 presenting with bladder dysfunction and compressive symptoms, though it was located intradurally rather than extradurally. Jungjun et al reported a large epidural CPPD mass mimicking a sequestered disc and causing cauda equina syndrome, sharing imaging and clinical features with our case.^15^ In contrast, Reis et al reported an incidental finding of an intramedullary mass later identified as tophaceous pseudogout, highlighting the variability in location and symptomatology.^16^ These comparisons reinforce the importance of including CPPD in the differential diagnosis of lumbar mass lesions with mass effect.

The American College of Rheumatology (ACR) and European Alliance of Associations for Rheumatology (EULAR) developed the 2023 ACR/EULAR classification criteria for CPPD.^1^ In patients with unexplained joint pain, swelling, or tenderness, crowned dens syndrome or calcium pyrophosphate crystals in synovial fluid confirm CPPD disease. If absent, a score above 56 points using a weighted criteria system, including clinical features, metabolic disorders, and lab/imaging results, can classify the patient as having CPPD.^1^ However, only symptomatic peripheral joints were considered in imaging criteria, complicating the diagnosis of spinal CPPD—apart from crowned dens syndrome. In our case, although synovial fluid analysis was not feasible due to the extradural location, the diagnosis was supported by histopathological confirmation of birefringent CPP crystals. Imaging revealed a mass-like lesion with features suggestive of calcification, but spinal findings are not included in the current classification system. As such, the patient did not meet the criteria through the formal scoring system, yet fulfilled diagnostic certainty via tissue analysis, underscoring the limitations of current criteria in atypical presentations such as spinal CPPD.

Radiographically, spinal CPPD can present a range of features, from simple calcium deposits to spinal masses, tophi, and hematomas, potentially causing compressive phenomena.^9^ A Combined Expert Committee recently established a consensus on the imaging feature criteria for CPPD.^4^ They identified suggestive indications of CPPD as linear or punctate opacities in the fibrocartilage or hyaline articular cartilage on conventional radiographs. These are distinguishable from the denser, nummular radiopaque deposits associated with basic calcium phosphate (BCP) deposition. Furthermore CPP crystals can be found in peri-articular structures (eg, cruciate ligament of the atlas), so location was not felt to be a definitive distinguishing feature. The Combined Expert Committee also established a consensus regarding the density specific to CPP deposits, recommending the use of cortical bone as a reference, as CPP is less dense and cortical bone is consistently visible on imaging. On conventional CT, CPPD typically presents as well-defined, linear, or punctate calcifications that are less dense than cortical bone and located within fibrocartilage or hyaline articular cartilage.^4^ A study evaluated the performance of the new definitions developed by the Combined Expert Committee for diagnosing CPPD in 67 knee osteoarthritis patients. While specificity was high (92%), sensitivity was low (54%). The definitions improve diagnostic specificity but cannot exclude the condition with negative findings.^17^ A recent meta-analysis confirmed that in peripheral joints, ultrasound is more sensitive than conventional radiography (85% vs 47%) while maintaining comparable specificity (87% vs 95%), supporting its complementary role in the diagnostic work-up of CPPD.^18^

Other useful imaging study for CPPD include dual-energy CT (DECT). Unlike standard CT, DECT enables in vivo discrimination of meniscal CPP deposits from calcium hydroxyapatite in subchondral and trabecular bone by assessing their unique attenuation and compositional properties.^19^ In contrast, MRI is not intended to characterize the types of calcium crystal deposits but rather to detect and quantify them.^4,6^ The agreed-upon definition for identifying CPPD on MRI includes “linear or punctate regions of low signal intensity located mainly in avascular white and red-white zones of menisci, and within hyaline cartilage surfaces, visible on dedicated specific sequences”. It might also show ligamentous thickening, vertebral endplate erosions, and abnormal signal intensity in adjacent vertebrae. Soft tissue masses show low to intermediate signal with minimal enhancement using gadolinium.^7^ However, MRI’s long acquisition time and complex data processing make it less ideal for evaluating CPPD.^4^

The current gold standard for diagnosing CPPD is identifying aggregates of basophilic to grey-brown material containing anisotropic rhombic to rod-shaped crystals with positive birefringence under polarized light (blue when they are parallel to the axis of the polarizer and yellow when they are perpendicular).^3^ These smaller, fainter crystals than urate crystals may also appear in mononuclear cells.^3,20^ Surrounding tissue often shows chronic inflammation, histiocytic reaction, giant cell reaction, chondrocyte hypertrophy, and myxoid degeneration.^20^

### Implications for Clinical Care

Spinal CPPD presents a diverse range of clinical manifestations, often resulting in underdiagnosis. Essential for identifying characteristic calcifications are diverse imaging techniques like conventional radiography, CT, and DECT. Diagnosis is definitively confirmed through histopathological identification of crystal aggregates. Clinicians should suspect CPPD in patients with suggestive calcifications and the presence of an intraspinal mass causing compression. Accurate diagnosis and comprehensive treatment strategies are crucial for effective management and improved patient outcomes. Treatment for CPPD primarily aims to control inflammation, as there are no treatments to dissolve the CPP crystals. This typically includes intra-articular glucocorticoids, oral colchicine, NSAIDs with gastroprotection, and low-dose prednisone. In refractory cases, hydroxychloroquine, methotrexate, or interleukin-1β inhibitors may be considered.^3^ Surgery may be required when conservative management fails or significant neurological compromise occurs due to tophaceous deposits causing compression, as in the present case, and should be tailored to the extent and location of the CPP deposition.^21^ Research highlights improved outcomes in spinal CPPD following surgical intervention without requiring total crystal removal, emphasizing a clinical presentation-based approach.^22^ The complexity of diagnosing and treating CPPD underscores the critical need for multidisciplinary collaboration. This case reinforces the importance of considering spinal CPPD in elderly patients with chronic back pain or spinal stenosis, particularly when imaging reveals calcified epidural or intradural masses. Although routine screening is not currently recommended, increased clinical awareness may prompt earlier diagnosis and avoid unnecessary delays in management.
